# Maximizing Room-Temperature Red Phosphorescence in Contorted Hexabenzocoronene Derivatives

**DOI:** 10.1021/acs.chemmater.6c00904

**Published:** 2026-05-20

**Authors:** Marko R. Ivancevic, Moses D. Ogbaje, Jesse A. Wisch, Daniel G. Oblinsky, Alice S. Fergerson, Emily C. Davidson, Gregory D. Scholes, Barry P. Rand, Chad Risko, Quinn C. Burlingame, Yueh-Lin Loo

**Affiliations:** † Department of Chemical and Biological Engineering, 6740 Princeton University, Princeton, New Jersey 08544, United States; ‡ Department of Chemistry & Center for Applied Energy Research, 4530 University of Kentucky, Lexington, Kentucky 40506, United States; § Department of Electrical and Computer Engineering, Princeton University, Princeton, New Jersey 08544, United States; ∥ Department of Chemistry, Princeton University, Princeton, New Jersey 08544, United States; ⊥ Andlinger Center for Energy and the Environment, Princeton University, Princeton, New Jersey 08544, United States

## Abstract

Organic molecules exhibiting second-scale room-temperature phosphorescence (RTP) in the red/near-infrared are particularly rare because low-energy excited states that are characteristic of these chromophores are susceptible to nonradiative deactivation. Here, we observe second-scale red RTP from contorted hexabenzocoronene (cHBC) embedded in a rigid polymer. This RTP is uniquely efficient as approximately 23% of the steady-state photoluminescence originates from triplets. We propose that this efficient triplet generation stems from intersystem crossing (ISC) that outcompetes symmetry-forbidden fluorescence. Density functional theory and time-dependent density functional theory calculations suggest that ISC occurs from the lowest energy singlet state of cHBC into a nearly resonant triplet state. Perdeuterating cHBC substitutes C–H stretches with lower-energy C–D stretches, which further suppresses nonradiative recombination and prolongs red RTP. The phosphorescence lifetime of perdeuterated cHBC-polymer composites exceeds 5 s, and has a steady-state phosphorescence fraction of 44%.

## Graphical abstract

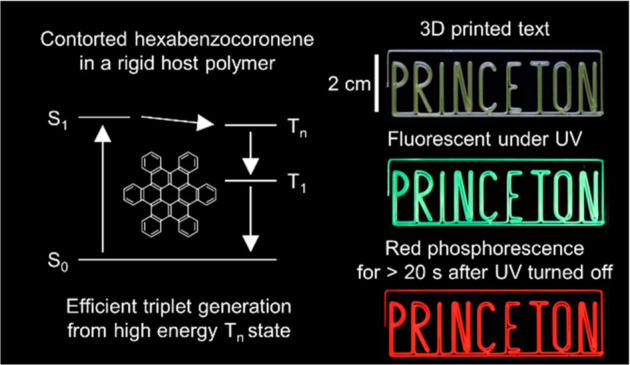

Organic molecules exhibiting second-scale red or near-infrared phosphorescence at room temperature (RT) are particularly desirable for bioimaging. This is because low-energy light effectively penetrates tissue samples and second-scale phosphorescence avoids competition with photoluminescence (PL) signatures that are present under excitation (e.g., environmental autofluorescence).^1,2^ Persistent second-scale emission can also be leveraged for authentication purposes in anticounterfeiting applications.^3^ However, organic phosphors with second-scale low-energy emission are rare.^4–8^ This is partially because low-energy excited states are more susceptible to nonradiative deactivation.^9^ Additionally, weak spin–orbit coupling in organic molecules leads to slow intersystem crossing (ISC) rates that cannot outcompete fluorescence. As a result, the lifetimes of red phosphorescent organic materials are typically on the order of a few ms up to several hundred ms.^10–15^ A particularly long lifetime of 2.8 s was observed in a system comprising coronene and perylene red blended in poly­(methyl methacrylate) (PMMA), but this emission requires the transfer of long-lived excitons from coronene to perylene red.^16^

We previously demonstrated second-scale RT phosphorescence (RTP) from a wide library of organic chromophores.^17^ Submicron chromophore aggregates dispersed in a rigid polymer matrix suppress molecular vibrations that can deactivate excitons, while minimizing triplet–triplet annihilation, and promoting excimer-mediated ISC in some cases. Among the chromophores initially screened, we found that only contorted hexabenzocoronene (cHBC) in PMMA exhibited RTP without the need for aggregation. However, inducing cHBC aggregation in PMMA further extends the RTP lifetime of these composites. While the submicron scale aggregates do not produce meaningful X-ray diffraction signal, polarized optical microscopy showed birefringence, which strongly suggests that the aggregates are crystalline.^17^

Here, we examine the photophysical properties of cHBC and find approximately 23% of the steady-state PL at RT originates from triplets. This relatively high triplet emission fraction suggests that cHBC’s ISC competes favorably with fluorescence and nonradiative recombination. The high molecular symmetry of cHBC (point group D_3h_) dramatically decreases the oscillator strength and rate of fluorescent transitions, enabling efficient ISC.^18–20^ We employ density functional theory (DFT) and time-dependent DFT (TDDFT) calculations and optical spectroscopy to further investigate the triplet generation mechanisms in cHBC. The DFT/TDDFT results indicate the presence of a higher energy triplet state (T_6_) with energy that is comparable to the lowest-lying singlet (S_1_) state. We hypothesize that the energetic resonance between these two states imparts faster ISC relative to the conventional T_1_ to S_1_ pathway. Finally, by partially or completely eliminating C–H stretching modes in cHBC via deuteration, we find a reduction in nonradiative recombination and an increase in RTP lifetime. With a lifetime of >5 s, perdeuterated cHBC exhibits the longest lifetime of organic red phosphors reported to-date.

The inset of Figure 1a shows cHBC’s molecular structure. Drop-cast films containing 0.2 wt % cHBC-in-polycarbonate (PC) were prepared on glass, then annealed above the *T*_g_ of PC prior to photophysical characterization. We use amorphous PC (*T*_g_ ∼150 °C) instead of PMMA (*T*_g_ ∼100 °C) because the higher *T*_g_ enables photophysical characterization of the composite in its glassy state at higher temperatures. As-cast films exhibit second-scale red RTP, as shown in Figure S1. Thermal annealing at 160 °C for 1 h induces aggregation of cHBC, which further suppresses nonradiative recombination and extends the RTP lifetime from 520 to 770 ms (Figure 1b). Previous studies on cHBC indicate that it can sustain temperatures greater than 400 °C without degradation.^21^

**Figure 1:**
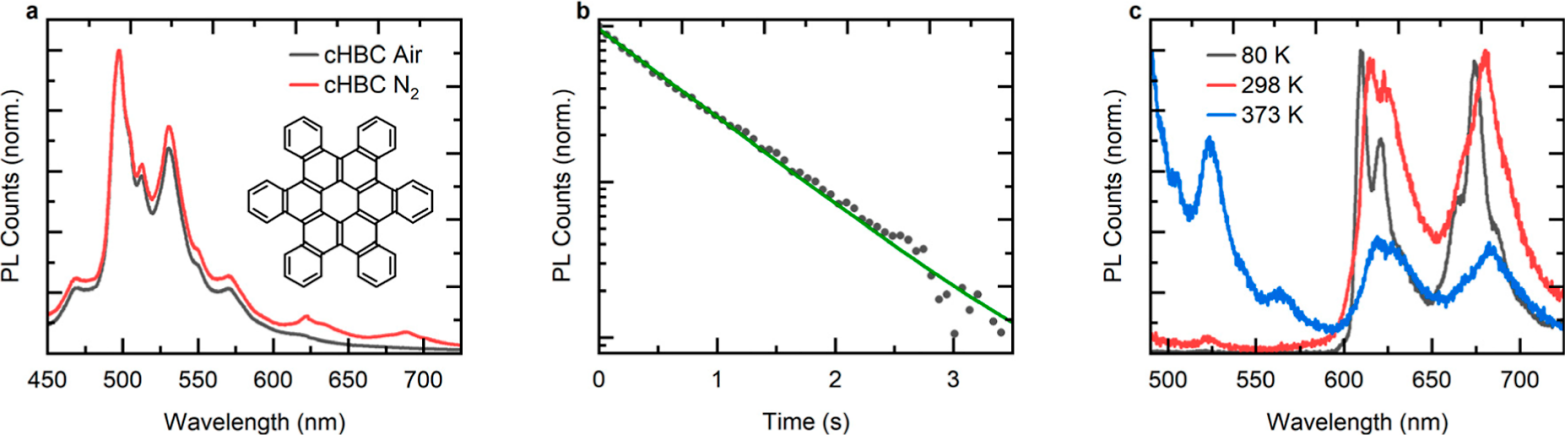
(a) Steady-state PL spectra of a 0.2 wt % cHBC-in-PC film in air and N_2_ at RT. Inset: cHBC’s molecular structure. (b) RT triplet PL transient (tracking emission at 620 nm) of a cHBC-in-PC film with an exponential fit. (c) Afterglow spectra of a cHBC-in-PC film at the indicated temperatures.

Figure 1a also shows the RT steady-state PL spectra of annealed cHBC-in-PC films in N_2_ and air. A red feature from 600 to 750 nm is observed when the experiment is carried out in N_2_. The absence of this red feature in the spectrum collected in air suggests oxygen quenching, which is indicative of phosphorescence. The phosphorescence of cHBC was directly measured at cryogenic temperatures in chloroform as shown in Figure S2. The overlap between the low-temperature cHBC emission in solution and cHBC-in-PC films in N_2_ further confirms that the film emission is phosphorescent.^9^ The fraction of steady-state emission attributable to cHBC triplets (*f*_T_) is determined to be 23% per eq 1

1 $$f_{T}=\frac{\sum {PL}_{N_{2}}-\sum {PL}_{Air}}{\sum {PL}_{N_{2}}}$$

where ∑PL_N2_ and ∑PL_Air_ are the integrated photon counts of the PL spectra collected in N_2_ and air, respectively, normalized to the emission peak at 497 nm.

Representative afterglow spectra (collected ∼20 ms after ceasing excitation) of this film at 80, 298, and 373 K are shown in Figure 1c. The afterglow spectrum at 80 K originates entirely from phosphorescence. At 298 K, the afterglow spectrum contains both phosphorescence and fluorescence signatures, while fluorescence is significantly more intense than phosphorescence in the afterglow spectrum at 373 K. We find that the singlet and triplet emission have nearly identical decay dynamics (Figure S3). These observations are consistent with singlet emission originating from thermally activated delayed fluorescence.^9,22–24^ We note that because the excitations responsible for TADF spend most of their time as triplets, TADF is also readily quenched by the presence of oxygen. The triplet fraction, *f*_T_, calculated above thus does not explicitly differentiate between phosphorescence and TADF.

The long phosphorescence lifetime can be attributed to the rigid confinement of cHBC in glassy PC, which effectively suppresses nonradiative recombination. Furthermore, the large steady-state triplet emission fraction suggests ISC is competitive with radiative singlet recombination.^25–28^ We conducted nanosecond time-resolved PL (TRPL) and PL quantum yield (PLQY) measurements to estimate the ISC rate of an as-cast cHBC-in-PC film (described in Methods). The singlet decay kinetics shown in Figure 2a were used to determine the singlet lifetime (τ_*S*_) and quantify the ISC rate intrinsic to cHBC-in-PC while minimizing potential excimer enhancement.^17,29^ Consider eqs 2–3 below

$$\Phi_{F}=\frac{k_{F}}{k_{F}+k_{nr}+k_{ISC}}=k_{F}\tau_{S}$$ 2

3 $$\Phi_{ISC}=k_{ISC}\tau_{S}\approx 1-\Phi_{F}$$

where Φ_F_ is the fluorescence quantum yield, *k*_F_ is the rate of fluorescence, *k*_nr_ is the rate of nonradiative recombination, *k*_ISC_ is the rate of ISC, and Φ_ISC_ is the ISC quantum yield. Using the measured values of Φ_*F*_ and τ_*S*_, we determine *k*_*F*_ ∼3.8 × 10^6^ s^–1^ from eq 2. Provided cHBC is a large aromatic molecule, we invoke Ermolaev’s rule (eq 3) to estimate *k*_ISC_ ∼2.1 × 10^7^ s^–1^ (all parameters summarized in Table S1 with additional details of the calculations).^25–28^ Our observation that *k*_ISC_ > *k*_*F*_ is consistent with the efficient triplet generation seen in this system.

**Figure 2:**
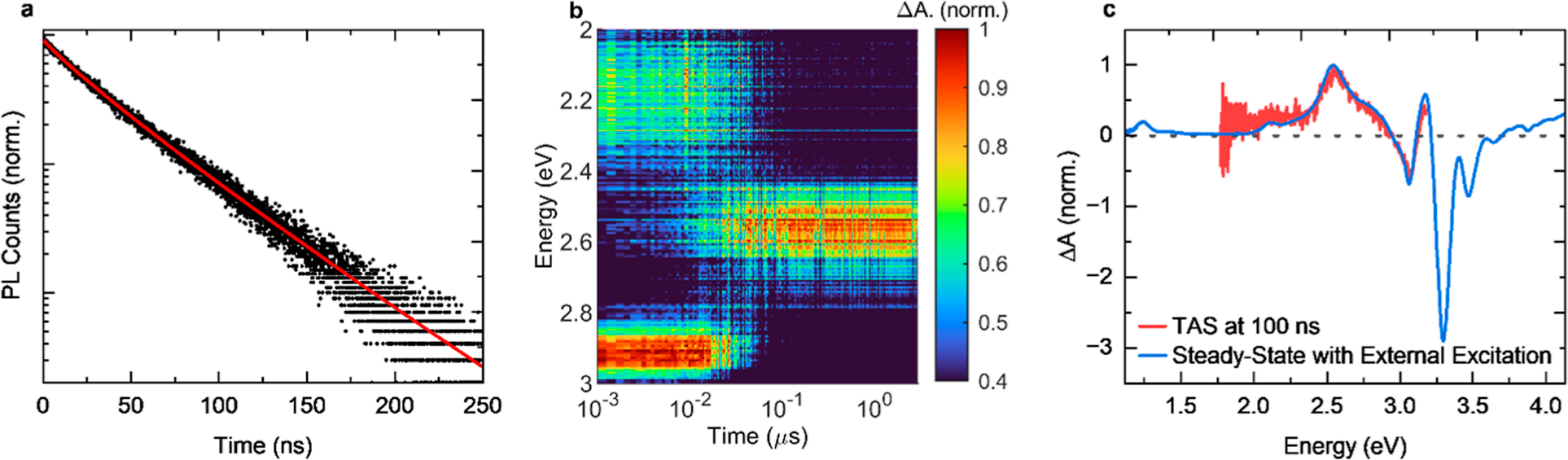
(a) Nanosecond time-resolved PL decay curve (tracking emission at 490 nm) of the as-cast 0.2 wt % cHBC-in-PC film with an exponential fit. (b) Transient absorption spectrogram of an unannealed cHBC-in-PC film showing features we ascribe to singlet absorption (∼2.8 to 3 eV and ∼2 to 2.4 eV) and triplet absorption (∼2.4 to 2.7 eV). (c) Steady-state absorption spectrum subtracted from the steady-state absorption under continuous external excitation from a 370 nm LED (steady-state with external excitation) and the ultrafast transient absorption spectrum obtained at 0.1 μs (TAS at 100 ns).

Reverse saturable absorption (RSA) can occur when the ground state of a material is partially depleted through the formation of a large population of excited states. Absorption by excited states (triplets in this case) into higher energy excited states thus becomes more common at the expense of ground state absorption events. These processes form the basis for ultrafast transient absorption spectroscopy. The ultrafast transient absorption spectrogram (Figure 2b) of an as-cast cHBC-in-PC film depicts singlet absorption features between 2.0 and 2.4 eV and 2.8–3.0 eV that decay over several tens of nanoseconds. Concomitant with this decay, we observe the growth of a triplet absorption feature between 2.4 and 2.7 eV. Once formed, this triplet absorption feature persists for the remainder of the 3 μs experiment—consistent with the second-scale triplet emission observed in these films. Organic molecules typically require high-intensity (∼GW/cm^2^) pulsed laser irradiation to induce RSA.^30,31^ However, the second-scale phosphorescence of cHBC allows RSA to be observed at steady-state under relatively weak (∼9.8 mW/cm^2^) continuous excitation from a 370 nm LED. Figure 2c shows the difference spectrum between the nonilluminated absorption spectrum of an as-cast cHBC-in-PC film and its absorption spectrum under continuous LED excitation. Overlaid in Figure 2c is the ultrafast transient absorption spectrum at 100 ns. Despite the dramatically different time scales and excitation intensities of the measurements, these spectra closely overlap when normalized. We attribute this similarity to triplets forming within ∼100 ns of excitation, and remaining in the film for several seconds, which allows a large steady-state triplet population to accumulate, even when they are excited by a weak source.

Calculations were performed (DFT and TDDFT as described in Methods) to identify the 10 lowest energy singlet and triplet states of cHBC (Tables S2 and S3). As illustrated in Figure 3, the TDDFT-calculated energy of T_6_ (2.78 eV) is < 0.1 eV lower than S_1_ (2.86 eV). This potential S_1_ → T_6_ pathway would produce faster ISC than S_1_ → T_1_ since ISC rates exponentially decrease as the exchange splitting between the initial and final states increases.^9^ Furthermore, the calculated ISC rate is comparable to those of polycyclic aromatic hydrocarbons with similar intermediate T_n_ states, and is significantly faster than those without such a T_n_.^32^ While this small gap between S_1_ and T_6_ suggests that T_6_ can act as an energetically accessible acceptor state for ISC, explicit spin–orbit coupling calculations are required to confirm the efficiency of this pathway. The TDDFT calculations indicate that the oscillator strength of the S_1_ → S_0_ transition is approximately 0, attributable to a symmetry-forbidden transition between the excited and ground states.^18–20^ Experimentally measured fluorescence rates of cHBC are much slower than conventional fluorophores due to its high molecular symmetry (point group D_3h_), consistent with this picture.

**Figure 3:**
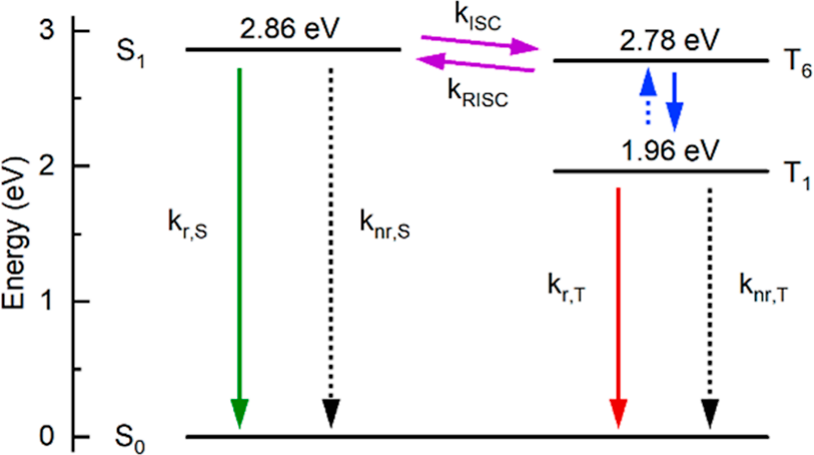
Jablonski diagram with DFT/TDDFT-calculated energy levels for cHBC illustrating the radiative and nonradiative recombination pathways of S_1_ and T_1_, internal conversion between T_6_ (shown due to its energetic proximity to S_1_) and T_1_, and ISC and reverse ISC between the singlet and triplet manifolds.

The C–H stretching modes in organic semiconductors can contribute significantly to nonradiative recombination because their high energy allows them to efficiently couple with excited states.^9,33^ To interrogate the impact of suppressing or eliminating these modes, we synthesized partially deuterated (d_16_-cHBC) and perdeuterated (d_24_-cHBC) cHBC analogs (described in Methods) with the molecular structures shown in Figure 4a. The Fourier transform infrared (FTIR) spectra of the compounds of interest in Figure 4b show decreasing intensity of C–H stretching modes (∼2800–3200 cm^–1^) and a concomitant increase in the intensity of C–D stretching modes (∼2200–2450 cm^–1^) with increasing deuteration. As a result of this suppression, drop-cast films containing 0.2 wt % of each deuterated analog in PC after annealing have considerably longer lifetimes of 2.5 and 5.1 s for d_16_-cHBC and d_24_-cHBC, respectively, as shown in Figure 4c.

**Figure 4:**
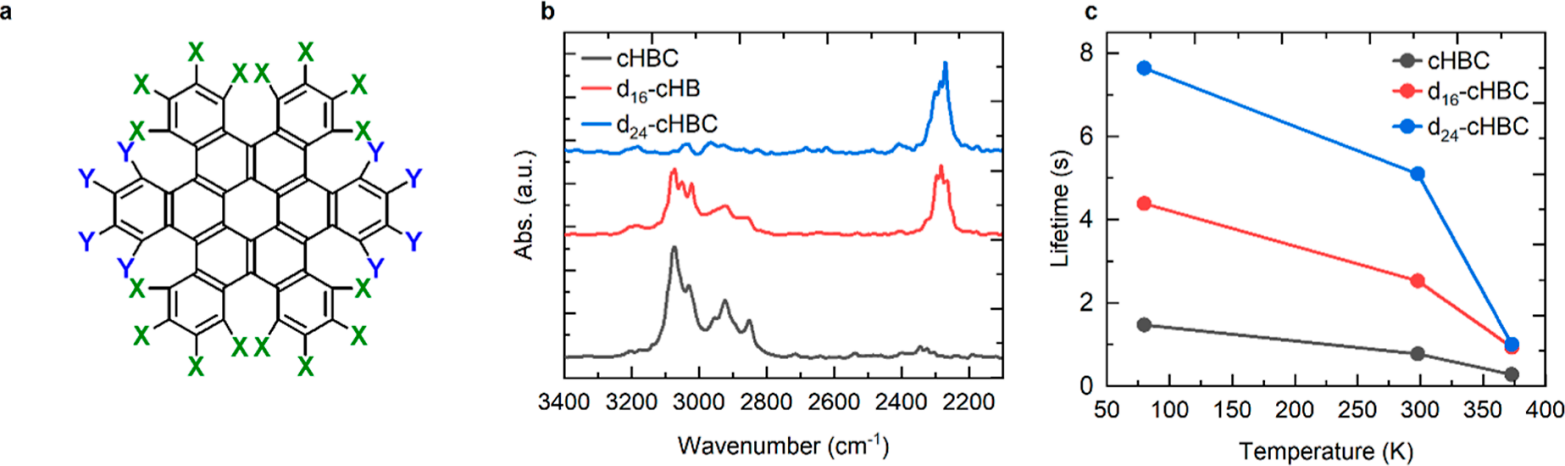
(a) Chemical structure of d_16_-cHBC (X = D Y = H) and d_24_-cHBC (X = Y = D). (b) FTIR spectra of cHBC, d_16_-cHBC and d_24_-cHBC highlighting the absorption features associated with C–H (∼2800–3200 cm^–1^) and C–D (∼2200–2450 cm^–1^) stretching modes. (c) Temperature-dependent phosphorescence lifetimes extracted from exponential fits to the transient PL data in Figures S3, S7–S8.

The TDDFT-calculated singlet and triplet energies of the deuterated analogs are identical to cHBC.^34^ We conducted nanosecond-scale TRPL and PLQY measurements on as-cast films of the deuterated analogs in PC (Figures S4 and S5) using an identical procedure to that used to assess ISC in cHBC-in-PC films. These data sets yield ISC rates of 1.8 × 10^7^ s^–1^ for d_16_-cHBC and 1.7 × 10^7^ s^–1^ for d_24_-cHBC, consistent with the notion that deuteration does not significantly alter the rate of ISC.^32^ Similar to the trends observed in cHBC, these ISC rates exceed the fluorescence rates (5.1 × 10^6^ s^–1^ and 4.9 × 10^6^ s^–1^ for d_16_-cHBC and d_24_-cHBC, respectively), and thus all three molecules should produce comparable triplet generation efficiencies. The considerably higher steady-state triplet emission fractions in d_16_-cHBC-in-PC (*f*_*T*_ = 33%) and d_24_-cHBC-in-PC (*f*_*T*_ = 44%) films are therefore attributed to the reduced nonradiative recombination rates in the deuterated molecules. Spectroscopic characterization of annealed d_16_-cHBC-in-PC and d_24_-cHBC-in-PC films are provided in Figures S6–S8.

Lastly, we 3D-printed 0.5 wt % d_24_-cHBC in polystyrene-poly­(ethylene-butylene)-polystyrene (SEBS) (described in Methods) to generate a skeletal representation of cHBC and “Princeton” text (Figure 5). The prints are off-yellow under ambient light but fluoresce green (450–600 nm) under continuous UV-illumination. After UV-illumination ceases, we observe red phosphorescence from the samples that persists for >20 s in N_2_, demonstrating the utility of this chromophore in creating customizable phosphorescent images/markers. We note that while the PS end blocks of SEBS are rigid, the triblock SEBS polymer is rubbery. This is consistent with the fact that d_24_-cHBC can phosphoresce even without a rigid polymer matrix, though preferential association with the rigid PS blocks may be occurring in this case.

**Figure 5:**
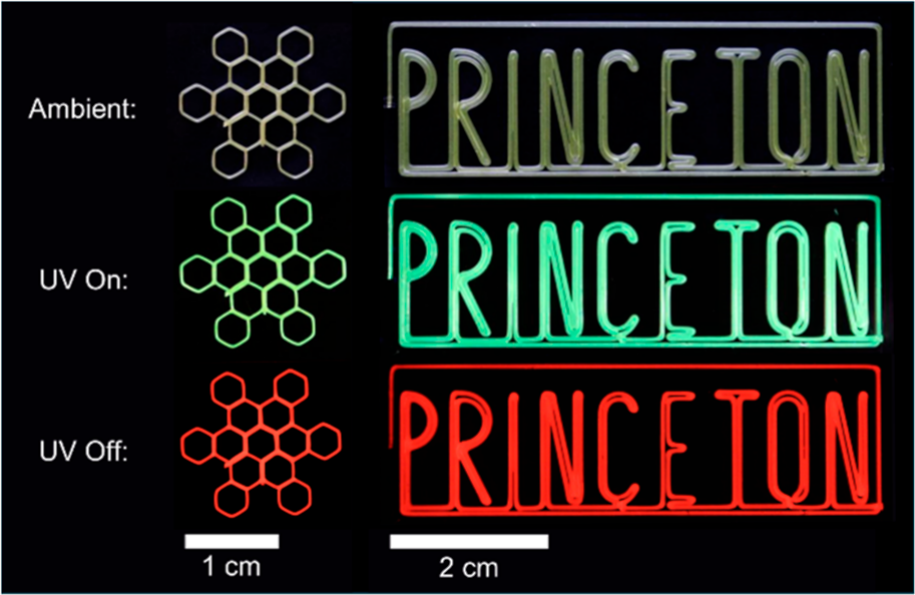
Photographs of a 3D-printed skeletal representation of cHBC and “Princeton” text using 0.5 wt % d_24_-cHBC in a polystyrene-poly­(ethylene-butylene)-polystyrene triblock copolymer under ambient light (ambient), in the dark with continuous UV excitation (UV On), and in the dark approximately 1 s after ceasing UV excitation (UV Off).

In summary, cHBC is an efficient red phosphor at RT when suspended in a PC matrix and can be made even more efficient by annealing above the *T*_g_ of PC to induce aggregation. We ascribe cHBC’s efficient triplet generation to its slow fluorescence and a TDDFT-calculated triplet state (T_6_) that is energetically resonant with S_1_. While cHBC aggregates in PC already exhibit exceptionally long phosphorescence lifetimes of 770 ms, we access molecules with phosphorescence lifetimes >5 s and steady-state triplet emission fractions exceeding 40% by eliminating C–H stretching vibrations through deuteration. The excitations of deuterated cHBC analogs are so long-lived that RSA can be observed in steady-state under weak LED emission with a comparable spectral signature to that of ultrafast transient absorption measurements. The highly suppressed nonradiative recombination in these materials also allows them to thermally repopulate singlet states at elevated temperatures and exhibit bright TADF. Finally, we have demonstrated the suitability of these uniquely long-lived red emitters with 3D printing. Further research could investigate applications in advanced bioimaging as well as phosphorescent markers and tracers for anticounterfeiting purposes with particular focus on preventing oxygen permeation that can quench triplets in air.

## Experimental Section

### Film Preparation

Glass slides (1.25 × 2.00 cm) were cleaned by sequential sonication in deionized water for 10 min, acetone for 5 min, and isopropyl alcohol for 5 min. Substrates were then dried with N_2_ and UV–ozone treated for 20 min prior to drop-casting. Separately, a 20 mg/mL stock solution of polycarbonate (PC) in chloroform was prepared. A 0.2 wt % relative to PC of cHBC (or deuterated analog) was added to a vial, followed by the appropriate volume of PC stock solution and allowed to stir until complete dissolution. The solutions were syringe filtered. Glass slides were placed on a hot plate set to 50 °C, and 60 μL of the solution was drop-cast onto the substrate. After complete evaporation of the solvent, optical measurements were conducted on the as-cast films. To induce aggregation of the chromophore, films were subjected to subsequent thermal annealing at 160 °C for 1 h.

### Second-Scale Time-Resolved Photoluminescence

“Afterglow” refers to the second-scale light emission that persists after ceasing excitation. Steady-state and afterglow photoluminescence spectra were collected using a SpectraPro HRS-300 spectrometer coulped with a PIX-400B CCD camera from Princeton Instruments, with a 370 nm long-pass filter placed prior to the spectrometer fiber couple. To collect steady-state spectra, we used a ThorLabs mounted 340 nm LED at an optical power density of approximate 60 μW/cm^2^ and 10 nm full width half-maximum bandpass filter. Afterglow spectra were collected by exciting the samples with a ThorLabs 370 nm mounted LED at a power density of about 135 mW/cm^2^ along with a collimator to focus the beam. The afterglow spectra of the molecules were collected in 1 PL day at a frame collection frequency of 15.3 Hz. A Linkam THMS600 temperature-control stage was used to control sample temperature and atmosphere (air or N_2_) during photoluminescence measurements. For measurements conducted in N_2_ the samples were loaded onto the stage, sealed, and purged with N_2_ for 10 min.

### Nanosecond-Scale Time-Resolved Photoluminescence

Nanosecond time-resolved photoluminescence decay curves were recorded using an FLS980 photoluminescence spectrometer (Edinburgh Instruments) with a 375 nm pulsed excitation source using a repetition rate of 2 MHz and a time-correlated single photon counting detector

### Transient Absorption Spectroscopy

Transient absorption experiments were conducted using a commercial spectrometer developed from Ultrafast Systems. The sample was excited with a 400 nJ pulse centered at 370 nm and generated from Ekspla PT403 ps laser. The probe pulse was generated using a Coherent Astrella (45 fs, 1 kHz, 5 W) by focusing an 800 nm fundamental into a vertically translated calcium fluoride crystal generating a supercontinuum. To minimize sample degradation, the sample was continuously translated, and dry nitrogen was flowed over the sample. Each sample was run for 40 min before sample degradation occurred. The resultant transient spectrum was background subtracted and analyzed using GloTarAn software package.^35^

### UV–Vis Absorption Spectroscopy

All steady-state UV–vis spectra were obtained using an Agilent 4853 UV–vis Spectroscopy System. A Kessil PR160–370 nm LED was used to externally excited the film.

### Density Functional Theory

DFT calculations were carried out with the ORCA program version 5.0.2.^36^ Optimization of the nondeuterated and deuterated cHBCs was performed using the ωB97X-D3 functional and the Def2SVP basis set.^37^ During the optimization process, dispersion corrections were accounted using Grimme’s D3 with BJ damping function.^38^ The range–separation parameter (ω) was tuned (Figure 1) for the nondeuterated cHBC structure to ensure that the vertical ionization potentials (IP) and the negative of the highest occupied molecular orbital (HOMO) energy are equal. This was done to satisfy Koopman’s theorem.^39^ The same ω-value obtained was used to optimize all structures since deuteration is not expected to considerably affect the electronic structure of the compound.^40,41^ Vibrational frequencies of the optimized geometries were carried out to ensure that the stationary point of each optimized structure is a true minimum on the potential energy surface and no imaginary frequencies are observable. Time dependent density functional theory (TDDFT) was then used for analysis of the excited state (singlet and triplet) energies as well as the absorption and emission spectra in gaseous phase of the three compounds at the same ωB97X-D3/Def2SVP level of theory. Optimization of the excited state structures of each structure was performed at the same level of theory.

### General Synthetic Information

All reagents were used as purchased without further purification. All reactions were run in oven-dried glassware. Flasks were fitted with a rubber septum and reactions were run under a positive pressure of nitrogen unless noted otherwise. Column chromatography was performed on a CombiFlash Rf system using RediSepTM normal phase silica columns (ISCO, Inc.) ^13^C NMR spectra were collected at room temperature on Bruker DRX spectrometers operating at 126 MHz. All peaks are reported as δ ppm. MALDI TOF/TOF were collected on a Bruker UltraFlextreme using an anthralin dithranol matrix. All FTIR spectra were collected on the Thermo Scientific Nicolet iN10 MX. Detailed synthetic information and characterization is available in the Supporting Information.

### 3D Printing

Polystyrene-*b*-poly­(ethylene-*co*-butylene)-*b*-pòlystyrene (SEBS) pellets (G1648, supplied by Kraton) were compounded with 0.5 wt % d_24_-cHBC by melt-pressing the two components between Teflon sheets at 150 °C, manually compressing the resulting sheet into a ball, and melt-pressing again a total of 8 times to ensure homogeneity. The mixture was 3D printed using a custom heated volumetric extruder mounted on Aerotech motion axes, using PVA-coated microscope slides as printing substrates. 3D printing was carried out with a nozzle temperature of 160 °C, a substrate temperature of 90 °C, a print height of 0.605 mm, and a print speed of 7.5 mm/s. 3D printing paths were designed manually and converted into Aerotech-executable files via custom Python scripts.

## Supplementary Material



## Notes

Full synthetic details, photoluminescence and absorption spectra, transient absorption spectra, and density functional theory calculated energy levels, are included in the Supporting Information which is available free of charge at (insert link) (PDF)
