# Supplementary material for: Maximizing Room-Temperature Red Phosphorescence in Contorted Hexabenzocoronene Derivatives
Source: Chem Mater. 2026 May 20;38(11):5753–9. doi: 10.1021/acs.chemmater.6c00904 (PMC13255170; doi:10.1021/acs.chemmater.6c00904)
Supplement: Supplementary file 1 [file cm6c00904_si_001.pdf]

Supporting Information

for

Maximizing room-temperature red phosphorescence in contorted hexabenzocoronene derivatives

Marko R. Ivancevic[1], Moses D. Ogbaje[2], Jesse A. Wisch[3], Daniel G. Oblinsky[4], Alice S. Fergerson[1], Emily C. Davidson[1], Gregory D. Scholes[4], Barry P. Rand[3,5], Chad Risko[2], Quinn C. Burlingame[1,], and Yueh-Lin Loo[1,*].*

[1]Department of Chemical and Biological Engineering, Princeton University, Princeton, NJ 08544, USA

[2]Department of Chemistry & Center for Applied Energy Research, University of Kentucky, Lexington, KY 40506, USA

[3]Department of Electrical and Computer Engineering, Princeton University, Princeton, NJ 08544, USA

[4]Department of Chemistry, Princeton University, Princeton, NJ 08544, USA

[5]Andlinger Center for Energy and the Environment, Princeton University, Princeton, NJ 08544 USA

*Correspondence: qb@princeton.edu and lloo@princeton.edu

Contorted hexabenzocoronene (cHBC), 6,13-bis(dibromomethylene)-6,13-dihydropentacene, and deuterated 1,2-bis(dibromomethyl)benzene were synthesized according to previously reported methods.[1,2]

Synthetic Procedures

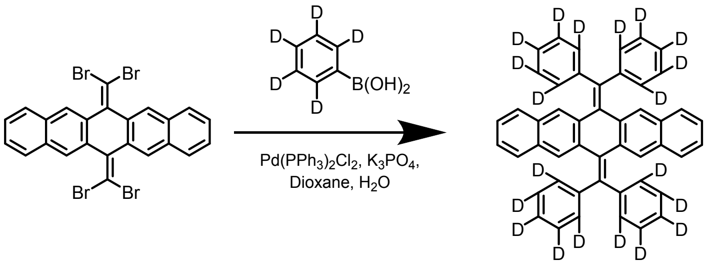

d_{20}-6,13-bis(diphenylmethylene)-6,13-dihydropentacene (1). To a dry vessel that had been evacuated with nitrogen was added 6,13-bis(dibromomethylene)-6,13-dihydropentacene (200 mg, 322 μmol), d_5-phenylboronic acid (204 mg, 1.61 mmol), K_3PO_4 (547 mg, 2.58 mmol), H_2O (2 mL) and dioxane (12 mL). The mixture was sparged with nitrogen for 15 minutes. Afterwards, $Pd(PPh_3)_2Cl_2$ was added (45 mg, 63.3 μmol) and the mixture was sparged with nitrogen for an additional 5 minutes. Then the reaction was heated to 80 °C and left to stir overnight. The reaction mixture was then transferred to a separatory funnel and worked up with water and DCM, and extracted with DCM two more times. The combined extracts were washed with brine, and dried over $MgSO_4$. The crude mixture was then concentrated via rotovap and purified by silica gel chromatography with 5:1 hexanes to DCM. The pure compound was an off-white solid (153 mg, 75% yield).

^{13}C NMR (126 MHz, $CDCl_3$): δ 142.27, 141.00, 136.05, 135.72, 131.57, 129.55, 129.37, 129.18, 128.22, 128.12, 127.93, 127.74, 127.17, 126.50, 126.32, 126.14, 125.63, 77.41, 77.16, 76.91.

MALDI TOF/TOF: m/z (M+) calculated 628.37, found 628.45.

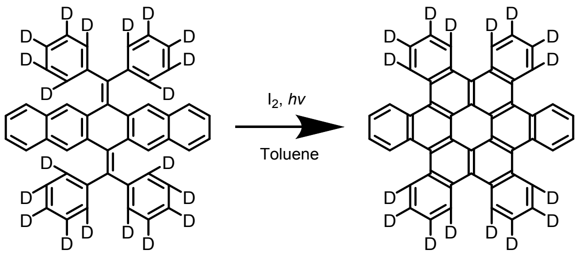

d_{16}-cHBC (2). **1** (100 mg, 159 μmol) was added to a 500 mL flat-bottomed flask with 200 mL of toluene and sonicated to facilitate dissolution. This mixture was then added to a photoreactor. Iodine (242 mg, 954 μmol) in 100 mL of toluene was added to the photoreactor and a nitrogen

bubbler was inserted to facilitate dissolution of all the reactants. The mixture was sparged with nitrogen for 20 minutes. 20 mL of propylene oxide was added and the UV lamp was turned on. The reaction was ran until the solution had turned yellow and exhibited a green fluorescence (this typically occurred after about 3 hours). The reaction mixture was then concentrated via rotovap to remove most of the solvent, leaving behind ~50 mL of solvent to prevent the product from sticking on the walls of the flask. Then, 300 mL of methanol was added to fully precipitate out the product. The product was then filtered and washed with more methanol, and then 100 mL of 2:1 hexane:DCM. The resulting yellow powder was dried under vacuum (82 mg, 83% yield).

^{13}C NMR (126 MHz, $CDCl_3$): Too insoluble to obtain.

MALDI TOF/TOF: m/z (M+) calculated 616.28, found 616.36.

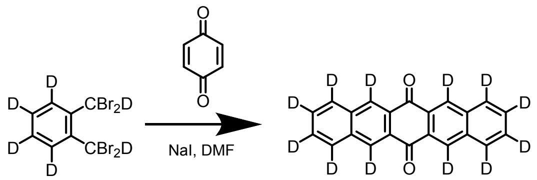

d_{12}-pentacene-6,13-dione (3). 1,2-bis(dibromomethyl)benzene (4.2 g, 9.82 mmol), benzoquinone (424 mg, 3.93 mmol), and NaI (3.68 g, 24.54 mmol) were dissolved in 20 mL of DMF. The mixture was sparged with nitrogen for 15 minutes. Then the mixture was heated to 110 °C overnight. The mixture was allowed to cool to room temperature and yellow solids appeared to precipitate out. 100 mL of methanol were added to precipitate the rest of the product out. The product was then filtered and washed with water and methanol, and dried under vacuum. A yellow product was obtained (921 mg, 29% yield).

^{13}C NMR (126 MHz): Too insoluble to obtain.

MALDI TOF/TOF: Did not ionize.

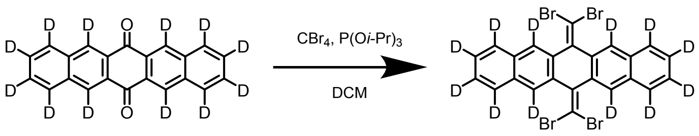

d_{12}-6,13-bis(dibromomethylene)-6,13-dihydropentacene (4). **3** (500 mg, 1.56 mmol) was added to a round-bottom flask with CBr_4 (3.1 g, 9.37 mmol) and 80 mL of DCM. The flask was cooled using an ice bath and sparged with nitrogen for 15 minutes. With the flask still in the ice bath, a solution of $P(O\text{-}iPr)_3$ (2.8 mL) in DCM (3.1 mL) was added dropwise. Once the addition was complete, the flask was removed from the ice bath and left stirring overnight. The reaction was quenched via the addition of 200 mL of methanol, at which point some product began

crystallizing out. The solution was concentrated via rotovap to remove most of the DCM, and left a suspension of crystals in methanol. The solids were filtered off and washed with more methanol and dried under vacuum. A white solid was obtained (740 mg, 76% yield) and used without further purification.

^{13}C NMR (126 MHz, CDCl$_3$): δ 140.04, 133.62, 132.06, 128.27, 128.08, 127.89, 127.42, 127.29, 127.23, 127.10, 127.03, 126.92, 90.94.

MALDI TOF/TOF: Did not ionize.

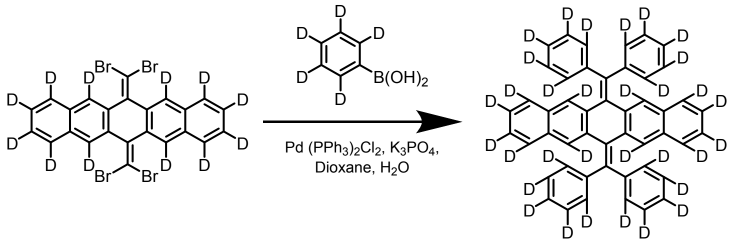

d$_{32}$-6,13-bis(diphenylmethylene)-6,13-dihydropentacene (5). To a dry vessel that had been evacuated with nitrogen was added **4** (200 mg, 316 μmol), d$_5$-phenylboronic acid (200 mg, 1.58 mmol), K_3PO_4 (537 mg, 2.53 mmol), H_2O (2 mL) and dioxane (12 mL). The mixture was sparged with nitrogen for 15 minutes. Afterwards, $Pd(PPh_3)_2Cl_2$ was added (44 mg, 63.3 μmol) and the mixture was sparged with nitrogen for an additional 5 minutes. Then the reaction was heated to 80 °C and left to stir overnight. The reaction mixture was then transferred to a separatory funnel and worked up with water and DCM, and extracted with DCM two more times. The combined extracts were washed with brine, and dried over $MgSO_4$. The crude mixture was then concentrated via rotovap and purified by silica gel chromatography with 5:1 hexanes to DCM. The pure compound was an off-white solid (145 mg, 71% yield).

^{13}C NMR (126 MHz, CDCl$_3$): δ 142.28, 140.98, 135.92, 135.66, 131.48, 131.40, 129.80, 129.67, 129.55, 129.36, 129.18, 128.46, 128.12, 127.94, 127.75, 127.55, 127.42, 127.23, 127.05, 126.96, 126.77, 126.59, 126.50, 126.31, 126.13, 125.29, 125.10, 124.93.

MALDI TOF/TOF: m/z (M+) calculated 640.45, found 640.55

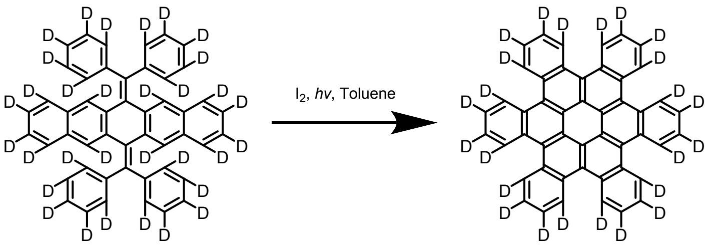

d$_{24}$-cHBC (6). **5** (100 mg, 156 μmol) was added to a 500 mL flat-bottomed flask with 200 mL of toluene and sonicated to facilitate dissolution. This mixture was then added to a photoreactor. Iodine (237 mg, 936 μmol) in 100 mL of toluene was added to the photoreactor and a nitrogen

bubbler was inserted to facilitate dissolution of all the reactants. The mixture was sparged with nitrogen for 20 minutes. 20 mL of propylene oxide was added and the UV lamp was turned on. The reaction was ran until the solution had turned yellow and exhibited a green fluorescence (this typically occurred after about 3 hours). The reaction mixture was then concentrated via rotovap to remove most of the solvent, leaving behind ~50 mL of solvent to prevent the product from sticking on the walls of the flask. Then, 300 mL of methanol was added to fully precipitate out the product. The product was then filtered and washed with more methanol, and then 100 mL of 2:1 hexane:DCM. The resulting yellow powder was dried under vacuum (84 mg, 86% yield).

^{13}C NMR (126 MHz, $CDCl_3$): Too insoluble to obtain.

MALDI TOF/TOF: m/z (M+) calculated 624.33, found 624.39.

Supporting Data

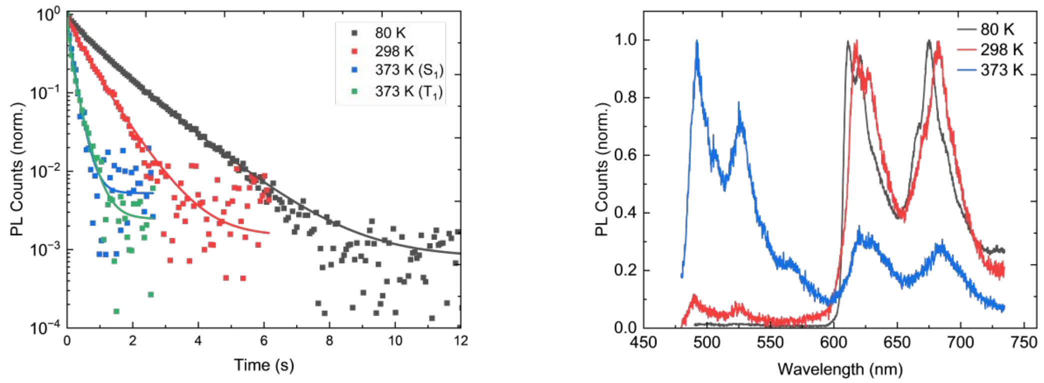

Figure S1. TRPL decay curve with exponential decay fits and afterglow spectra for as-cast 0.2 wt% cHBC-in-PC at the temperature indicated in the legend. We monitored 620 nm for the 80 K, 298 K, and 373 K (T_1) traces, and 505 nm for the 373 K (S_1) trace.

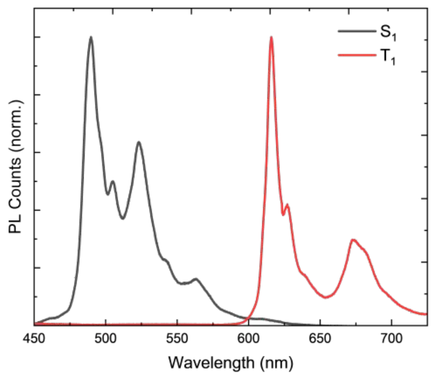

Figure S2. Steady-state photoluminescence of cHBC in chloroform at room temperature depicting singlet emission (S_1), and afterglow of that same solution at 80 K depicting triplet emission (T_1).

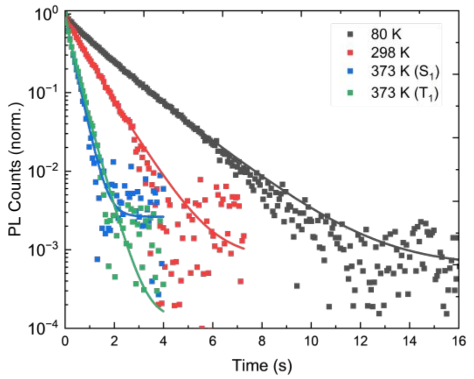

Figure S3. TRPL decay curve with exponential decay fits for 0.2 wt% cHBC-in-PC after annealing above T_g for one hour; spectra obtained at the temperature indicated in the legend. We monitored 620 nm for the 80 K, 298 K, and 373 K (T_1) traces, and 505 nm for the 373 K (S_1) trace.

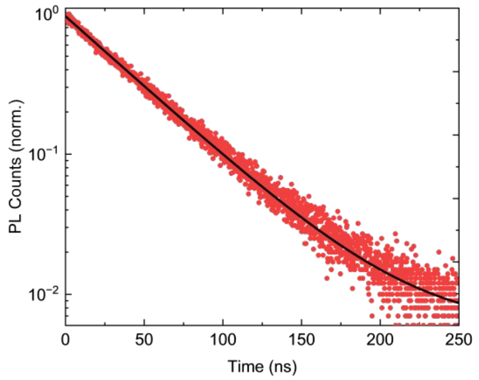

Figure S4. Nanosecond TRPL decay curve of the 0.2 wt% cHBC-in-PC after annealing above T_g with exponential decay fit. We monitored 490 nm to produce this trace.

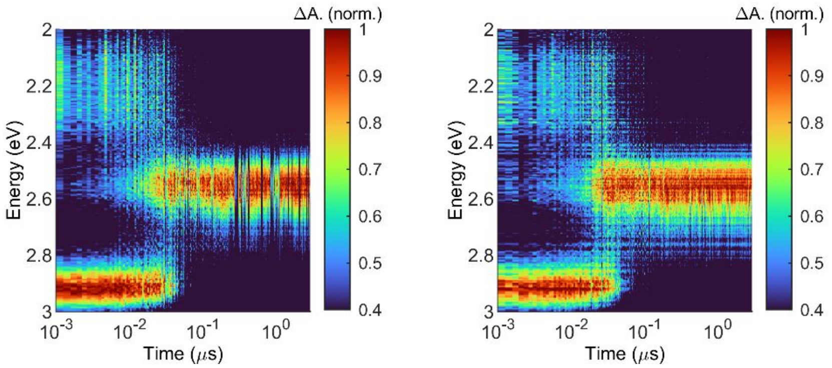

Figure S5. Transient absorption spectrogram for d_{16}-cHBC-in-PC (left) and d_{24}-cHBC-in-PC (right) used to extract rates of intersystem crossing.

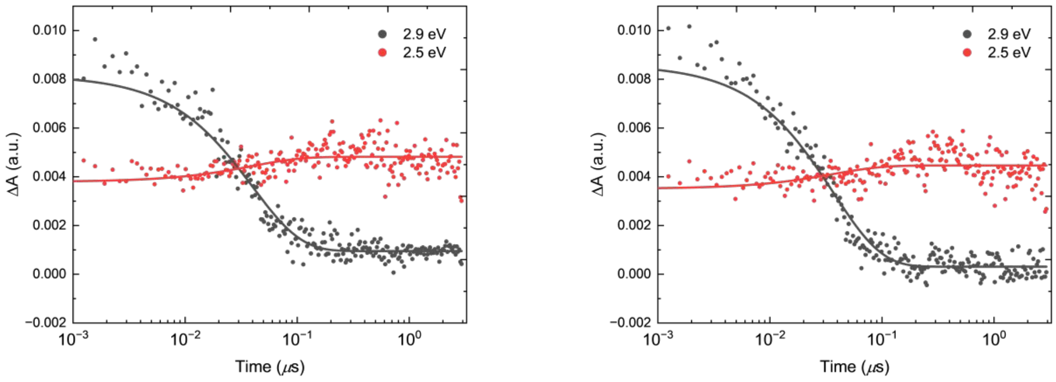

Figure S6. Representative absorption transients for 0.2 wt% d_{16}-cHBC-in-PC (left) and 0.2 wt% d_{24}-cHBC-in-PC (right).

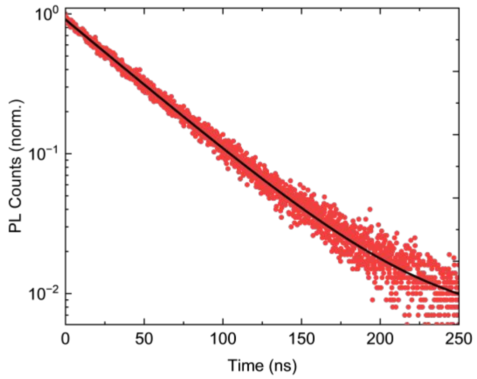

Figure S7. Nanosecond TRPL decay curve of the 0.2 wt% d_{16}-cHBC-in-PC after annealing above T_g with exponential decay fit. We monitored 490 nm to produce this trace.

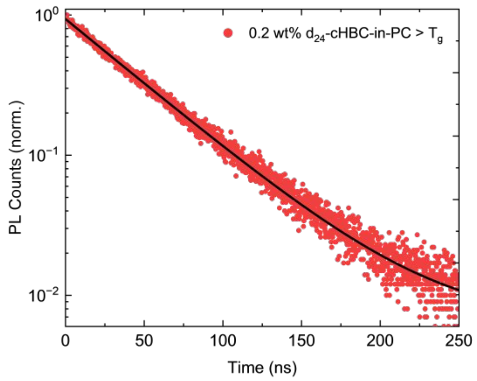

Figure S8. Nanosecond TRPL decay curve of the 0.2 wt% d_{24}-cHBC-in-PC after annealing above T_g with exponential decay fit. We monitored 490 nm to produce this trace.

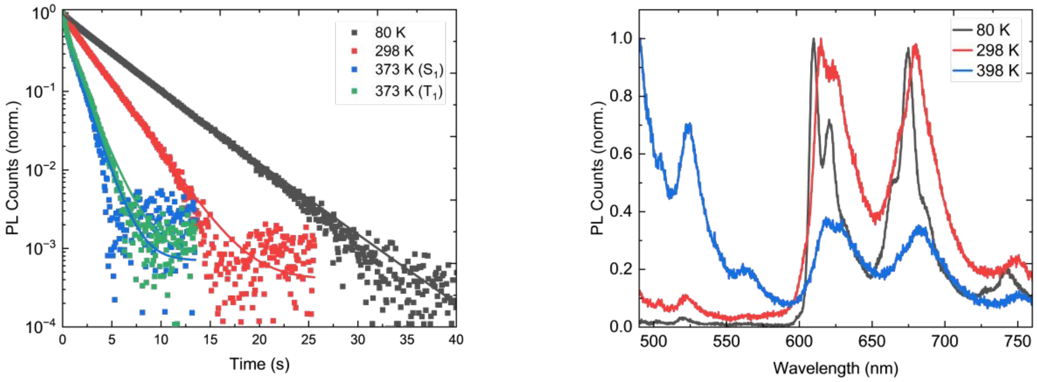

Figure S9. TRPL decay curve with exponential decay fits and afterglow spectra for 0.2 wt% d_{16}-cHBC-in-PC after annealing above T_g for one hour; spectra obtained at the temperature indicated in the legend. We monitored 620 nm for the 80 K, 298 K, and 373 K (T_1) traces, and 505 nm for the 373 K (S_1) trace.

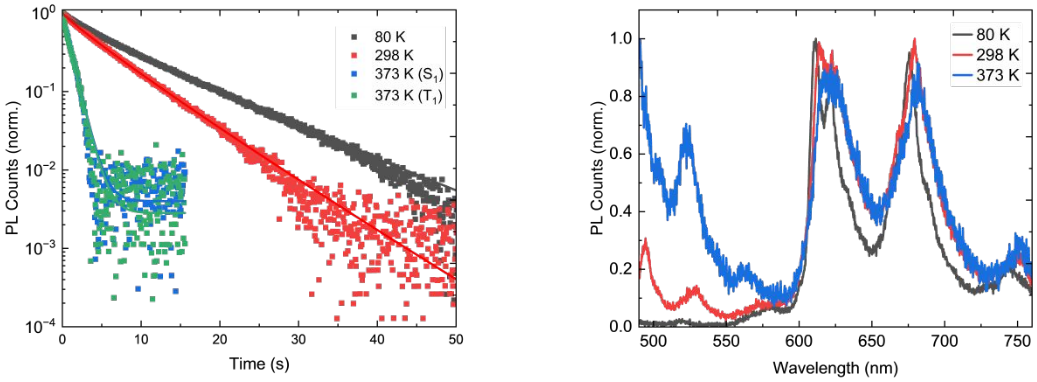

Figure S10. TRPL decay curve with exponential decay fits and afterglow spectra for 0.2 wt% d_{24}-cHBC-in-PC after annealing above T_g for one hour; spectra obtained at the temperature indicated in the legend. We monitored 620 nm for the 80 K, 298 K, and 373 K (T_1) traces, and 505 nm for the 373 K (S_1) trace.

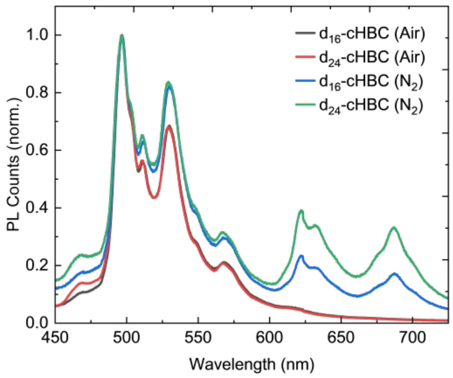

Figure S11. Steady-state PL spectra of a 0.2 wt% d_{16}-cHBC and d_{24}-cHBC in PC films in both air and N_2 at room temperature.

Table S1. TDDFT-derived energies, wavelengths, and oscillator strengths of the first 10 singlet excited states. These values are identical for cHBC, d_{16}-cHBC, and d_{24}-cHBC.

State	Energies (eV)	Wavelength (nm)	Oscillator Strength (a.u.)
1	2.86	433.4	0.0088
2	3.00	413.3	0.013
3	3.37	367.4	0.018
4	3.42	362.3	0.039
5	3.48	356.3	0.018
6	3.51	353.2	0.88
7	3.51	353.3	1.05
8	3.54	350.3	0.20
9	3.92	316.1	0.0064
10	4.18	296.5	0.0069

Table S2. TDDFT-derived energies and wavelengths of the first 10 triplet excited states. These values are identical for cHBC, d_{16}-cHBC, and d_{24}-cHBC.

State	Energies (eV)	Wavelength (nm)
1	1.96	632.1
2	2.45	505.5
3	2.48	498.4
4	2.70	459.3
5	2.70	458.7
6	2.78	446.2
7	3.30	375.5
8	3.37	368.1
9	3.39	366.6
10	3.46	358.4

Table S3. Summary of relevant photophysical parameters.

	$\tau_F\ (ns)$	ϕ_F	$k_F\ (s^{-1})$	$k_{ISC}\ (s^{-1})$
cHBC	40	0.15	$3.8 \cdot 10^6$	$2.1 \cdot 10^7$
d_{16}-cHBC	43	0.22	$5.1 \cdot 10^6$	$1.8 \cdot 10^7$
d_{24}-cHBC	45	0.22	$4.9 \cdot 10^6$	$1.7 \cdot 10^7$

Where the parameters are defined as
τ_F – Fluorescence lifetime
ϕ_F – Fluorescence quantum yield
k_F – Rate of fluorescence
k_{ISC} – Rate of intersystem crossing

Synthetic Characterization

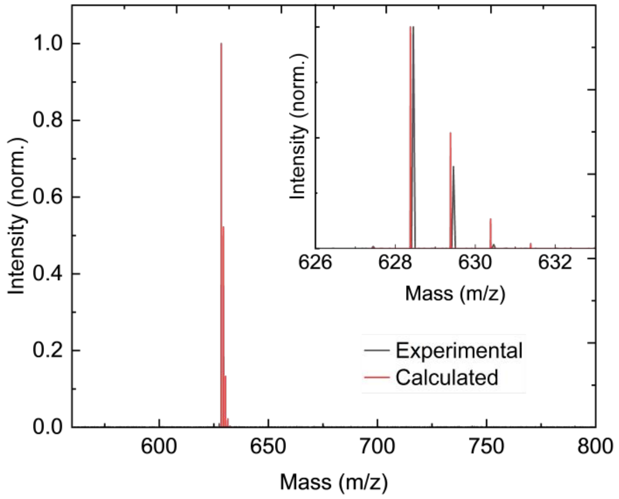

Figure S12. MALDI TOF spectrum of **1**.

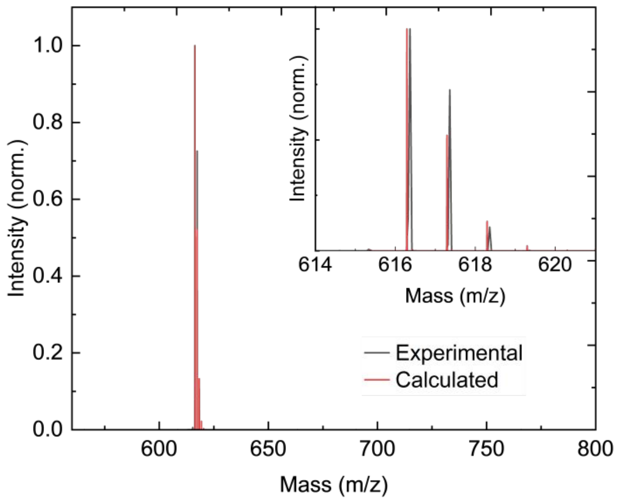

Figure S13. MALDI TOF spectrum of d_{16}-cHBC.

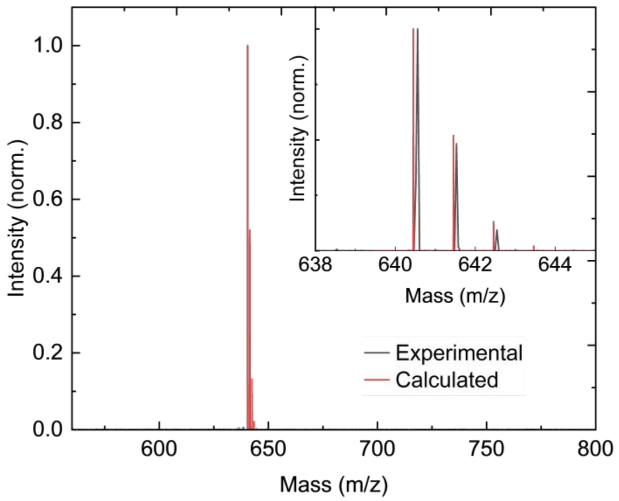

Figure S14. MALDI TOF spectrum of **5**.

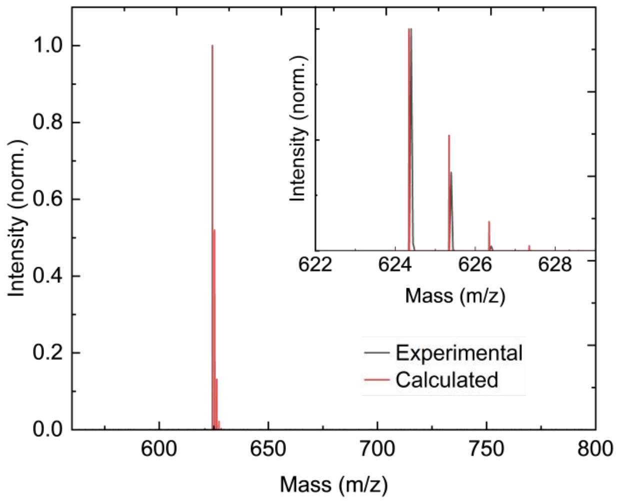

Figure S15. MALDI TOF spectrum of d_{24}-cHBC.

Supplemental References

(1) Davy, N. C.; Man, G.; Kerner, R. A.; Fusella, M. A.; Purdum, G. E.; Sezen, M.; Rand, B. P.; Kahn, A.; Loo, Y.-L. Contorted Hexabenzocoronenes with Extended Heterocyclic Moieties Improve Visible-Light Absorption and Performance in Organic Solar Cells. *Chem. Mater.* **2016**, *28* (2), 673–681.

(2) Barton, J. W.; Howard, J. A. K.; Shepherd, M. K.; Stringer, A. M. The Rearrangement of a Tetrahydrobiphenylene Derivative to a Bridged Benzocycloheptene. *J. Chem. Soc. Perkin 1* **1987**, No. 0, 2443–2445.
